# Characterization of surface proteins of *Cronobacter muytjensii *using monoclonal antibodies and MALDI-TOF Mass spectrometry

**DOI:** 10.1186/1471-2180-11-148

**Published:** 2011-06-25

**Authors:** Ziad W Jaradat, Abrar M Rashdan, Qotaiba O Ababneh, Saied A Jaradat, Arun K Bhunia

**Affiliations:** 1 Department of Biotechnology and Genetic Engineering, P. O Box 3030, Jordan University of Science and Technology, Irbid 22110, Jordan; 2 Department of Biochemistry and Biophysics, Texas A & M University, College Station, Texas 77843-2128, USA; 3 Princes Haya Biotechnology Center, Jordan University of Science and Technology, Irbid, 22110, Jordan; 4 Department of Food Science, Purdue University, West Lafayette, Indiana, 47907, USA

## Abstract

**Background:**

*Cronobacter *spp. is a newly emerging pathogen that causes meningitis in infants and other diseases in elderly and immunocompromised individuals. This study was undertaken to investigate surface antigenic determinants in *Cronobacter *spp. using monoclonal antibodies (MAbs) and MALDI-TOF Mass spectrometry.

**Results:**

Spleenocytes from mice that were immunized with heat-killed (20 min, 80°C) *Cronobacter *cells were fused with SP2 myeloma cells. Five desirable MAbs (A1, B5, 2C2, C5 and A4) were selected. MAbs A1, B5, 2C2 and C5 were of IgG2a isotype while A4 was an IgM. Specificity of the MAbs was determined by using immunoblotting with outer membrane protein preparations (OMPs) extracted from 12 *Cronobacter *and 6 non-*Cronobacter *bacteria. All MAbs recognized proteins with molecular weight ranging between 36 and 49 kDa except for one isolate (44) in which no OMPs were detected. In addition, MAbs recognized two bands (38-41 kDa) in four of the non-*Cronobacter *bacteria. Most of the proteins recognized by the MAbs were identified by MALDI-TOF peptide sequencing and appeared to be heterogeneous with the identities of some of them are still unknown. All MAbs recognized the same epitope as determined by an additive Index ELISA with their epitopes appeared to be conformational rather than sequential. Further, none of the MAbs recognized purified LPS from *Cronobacter *spp. Specificity of the MAbs toward OMPs was further confirmed by transmission electron microscopy.

**Conclusions:**

Results obtained in this study highlight the immunological cross-reactivity among *Cronobacter *OMPs and their *Enterobacteriaceae *counterparts. Nevertheless, the identity of the identified proteins appeared to be different as inferred from the MALDI-TOF sequencing and identification.

## Background

*Cronobacter *spp. (formerly *Enterobacter sakazakii*) is a non-spore forming, motile, facultative anaerobic Gram-negative bacillus and belongs to family *Enterobacteriaceae *[1,2]. Initially isolates of *Cronobacter *spp. (*Cronobacter*) were identified as yellow pigment producing *Enterobacter cloacae*. Later, Farmer et al., [3] reclassified them as a new species and were given the name *sakazakii *based on DNA-DNA homology, antibiotic susceptibility patterns and certain unique biochemical characteristics such as catalase production, the absence of oxidase and the production of yellow pigment in all tested strains.

More recent studies utilizing full length 16S rRNA gene sequencing, ribotyping, fluorescent-amplified fragment length polymorphism and DNA-DNA hybridization have demonstrated that *Cronobacter *is a heterogenic genus exhibiting a high degree of genetic and phenotypic diversity among species and comprises six species: *C. muytjensii, C. sakazakii, C. malonaticus, C. turicensis, C. dublinensis *and *C. genomospecies *I [4-7]. *Cronobacter *is considered an emerging pathogen; though, little is known about its virulence properties and antigenic determinants [8]. Recently, several studies have reported the involvement of an outer membrane protein (OMP), OmpA, in pathogenesis of *C. sakazakii*; however, nothing is known about its antigenicity. Besides, little is known about OMPs from other *Cronobacter *species [8-10]. In contrast, the virulence and antigenic properties of OMPs of closely related *Enterobacter *species including *E. aerogenes *[11] and *E. cloacae *[12,13] were studied well.

Prematurely born infants with low birth weights and infants in neonatal intensive care units are highly susceptible to *Cronobacter *infections with the pathogen being transmitted primarily from contaminated environments to the infant formula during the preparation [14-20]. In rare cases, nosocomial infections can happen in adults especially in immunocompromised ones [21]. In 2004, a joint FDA/WHO workshop raised an alert concerning the presence of *Cronobacter *in powdered infant formula (PIF) and recommended applying higher microbiological standards during its manufacturing [22]. This warning culminated into increased research efforts to study *Cronobacter *including the development of improved isolation and identification methods, and understanding of the growth and survival characteristics.

Antibodies are the most frequently used tools to study bacterial antigenic determinants; however, little is known about the production of monoclonal antibodies that recognize *Cronobacter *antigenic determinants. In this paper we describe the production and characterization of 5 MAbs that recognize outer membrane proteins of *Cronobacter*. In addition, antigenic properties, identification, distribution and cell surface localization of the MAbs- recognized OMPs were examined using electron microscopy and MALDI-TOF spectrometry. To our knowledge, this is the first report on using monoclonal antibodies to study the surface antigens of this pathogen.

## Methods

### Materials

Alkaline phosphatase-conjugated goat anti-mouse immunoglobulin, complete Freund's adjuvant, incomplete Freund's adjuvant, sarkosyl, DMSO, pancreatic RNase and DNase and a mouse subisotyping kit were from Sigma-Aldrich, USA. Gold-conjugated (18 nm) anti-mouse IgG was obtained from Jackson Immunochemicals, USA. Polyethelyene glycol 4000 was from Fluka, USA. Micro test plates, tissue culture plates and flasks were from Griener, Germany. Coommassie Brilliant blue G-250 was from BDH chemicals, Ireland and BSA was from Biobasic, Canada; Proteinase K was from Promega, USA. Goat anti-mouse-conjugated to horse radish peroxidase (HRP) was from Santa Cruz, USA. Penicillin, streptomycin and amphotercin B were from PAA Laboratories GMBH, Austria. Recovery cell culture freezing media was from Gibco, USA. Myeloma SP2 cells were a gift from Dr. Khalid Qaoud, Yarmouk University, Jordan. All other chemicals and reagents were of analytical grade.

### Bacteria and growth conditions

Stock cultures were maintained through out this study on Trypticase Soy Agar (TSA) (Oxoid, UK) or nutrient agar plates (HiMedia, India) at 4°C until use. The type strain *C. muytjensii *ATCC 51329 was grown overnight at 37°C in nutrient broth (NB) (HiMedia). Cells were harvested by centrifugation (5,000 × g, 10 min), washed twice with 0.1 M PBS (pH 7.2) and adjusted to 10^8 ^CFU ml^-1 ^using McFarland standard. Bacterial cells were heated at 80°C for 20 min in a water bath and were subsequently used for immunization of mice and screening of hybridoma cells for MAbs production using ELISA. Several *Cronobacter *strains used in the study were isolated from Jordan (Table 1). These isolates were identified and characterized by several traditional and molecular methods [19]. The 16S rRNA sequences of the isolates were deposited in the GenBank (MD, USA) (Table 1), while the isolates were deposited in the Egyptian Microbial Culture Collection (Ain Shams University, Cairo, Egypt).

**Table 1:** *Cronobacter *and Non-*Cronobacter *strains used in this study.

Isolate #	Isolate identity	Source	Isolate ID	GenBank ID based on 16S rRNA sequence
-	*C. muytjensii*	-	ATCC 51329	-
C4	*C. sakazakii*	Clinical	-	
C6	*C. sakazakii*	Clinical	CDC 407-77	-
C13	*C. sakazakii*	Clinical	ATTC 29004	-
Jor* 44	*C. sakazakii*	Food	EMCC 1904	FJ906902
Jor* 93	*C. sakazakii*	Food	EMCC1905	FJ906906
Jor* 112	*C. muytjensii*	Food	EMCC1906	FJ906909
				
Jor* 146_A_	*C. sakazakii*	Food	EMCC1907	FJ906897
Jor* 146_B_	*C. sakazakii*	Food	EMCC1908	FJ906910
Jor* 149	*C. muytjensii*	Food	EMCC1909	FJ906912
				
Jor* 160_A_	*C. sakazakii*	Environment	EMCC1910	FJ906914
Jor* 170	*C. turicensis*	Food	EMCC1912	FJ906916
				
None -*Cronobacter*				
-	*C. freundii*	-	ATCC 43864	-
-	*E. coli*	-	ATCC 35218	-
-	*L. ivanovii*	-	ATCC 19119	-
-	*P aeruginosa*	-	ATCC 27833	-
-	*S. enterica Choleraesuis*	-	CIP 104220	-
-	*S. sonnei*	-	ATCC 9290	-

Jor*: Strains were isolated from food and environmental samples collected in Jordan and were deposited in the Egyptian Microbial Culture Collection (EMCC; Ain Shams University, Cairo, Egypt) and their 16S rRNA sequences were deposited in the GenBank. C: clinical samples isolated from patients obtained from CDC (Atlanta, GA, USA) and were a gift from Dr. Ben Davies Tall from U.S. FDA. All the other isolates were obtained from the American Type Culture Collection (ATCC) except for *Salmonella *which obtained from the Collection of Institute Pasteur (CIP) and *S. sonnei *which was a local strain

### Lipopolysaccharide (LPS) extraction and antigen preparation

LPS was prepared following the method described by Jaradat and Zawistowski [23], with minor modifications. Briefly, *C. muytjensii *ATCC 51329 cells were harvested from an overnight culture by centrifugation (5,000 × g, 10 min) and resuspended in 50 ml of 50 mM sodium phosphate buffer, pH 7.0. The cells were sonicated 5 times for 45 s intervals at 300 Watts (Branson Sonifier). The sonicated suspension was incubated with pancreatic RNase and DNase (0.1 μg ml^-1^) in 20 mM MgCl_2 _at 37°C for 10 min, followed by 10 min at 60°C and then mixed with an equal volume of preheated 90% phenol. Following incubation (70°C for 15 min) with occasional mixing, the mixture was centrifuged (18,000 × g, 1 h) and the resulting aqueous layer was collected and dialyzed using dialysis tubing of 6,000-8,000 MW cutoff at room temperature against several changes of distilled water until no detectable phenol odor remained. The dialysate was treated with 20 μg/ml of Proteinase K in 0.1 M Tris-HCl (pH 8.0) at 60°C for 1 h followed by overnight incubation at 37°C. The samples were then lyophilized and stored at -20°C until used. For antigen preparation, the extracted LPS from *Cronobacter *was mixed (1:1) with 30% (w/v) polyacrylamide solution; ammonium persulfate (50 μl) and TEMED (10 μl) were added to the mixture to obtain a 15% polyacrylamide gel (v/v) [24]. The gel-containing LPS was frozen in liquid nitrogen and ground with a pestle and mortar into a fine powder. The powder was dissolved in 10 ml PBS (0.1 M, pH 7.0) and immediately used for immunization [25].

### Outer membrane protein extraction

OMPs were extracted using the sarkosyl-based method described by Davies et al., [26]. Briefly, *Cronobacter *cells were harvested from overnight cultures by centrifugation, and then treated with 0.1 μg of bovine RNase and DNase in 20 mM MgCl_2 _for 10 min at 37°C. Next, the cells were sonicated for 10 min in 45 sec intervals at 300 watts on crushed ice and were centrifuged (5,000 × g for 30 min at 4°C). The supernatant was collected and re-centrifuged (29,000 × g for 2 h at 4°C). The resulting pellet was treated with 10 ml of 2% (w/v) sarkosyl for 30 min at room temperature. The mixture was centrifuged (29,000 × g for 2 h). The resulting pellet was washed with 10 ml of 20 mM Tris-HCl (pH 7.7) containing 2% (w/v) SDS and re-centrifuged (29,000 × g for 2 h at 4°C). The final pellet, which contained OMPs, was resuspended in distilled water, aliquoted and stored at -20°C for further use.

### Production of monoclonal antibodies against *Cronobacter *spp

Female Balb/c mice (6 to 8 weeks old) were initially immunized intraperitoneally with 200 μl (10^8 ^CFU ml^-1^) of heat-killed bacterial suspension (*C. muytjensii *ATCC 51329) mixed with complete Freund adjuvant at a 1:1 ratio. Subsequently, 4 booster doses were administrated at weekly intervals using the same amount of immunogen but prepared with incomplete Freund adjuvant. Simultaneously, female Balb/c mice (6 to 8 weeks old) were immunized intraperitoneally with 200 μl of polyacrylamide-LPS preparation in PBS for at least 8 wks at weekly intervals. Myeloma SP2 cells were maintained in RPMI media supplemented with 10% Fetal Calf Serum (FCS), 20 U of penicillin, 20 U streptomycin and 2.5 μg ml^-1 ^amphotercin B. At the day of fusion, the actively grown myeloma culture was washed twice using serum-free media (SFM) and adjusted to the desired concentration. The fusion was performed according to the method described by Liddell and Cryer [27] using 40% (w/v) polyethylene glycol 4000 as the fusing agent in sterile SFM adjusted to pH 7.4. Spleen cells harvested from immunized mice and myeloma cells were fused at a ratio of 8:1. Two weeks later, hybridomas were screened for the production of MAbs by ELISA using heat killed *Cronobacter *and non-*Cronobacter *cells. Hybridomas reacting specifically with *Cronobacter *were expanded and cloned at least three times by limiting dilution. Positive clones were frozen in recovery cell culture freezing media^® ^or FCS supplemented with 4% (v/v) DMSO and stored at -80°C overnight before being transferred to liquid nitrogen. The positive clones were propagated either in tissue culture or by ascitic fluid using the procedure of Harlow and Lane [28]. Isotypes of purified monoclonal antibodies from ascites or spent medium were determined using the mouse type subisotyping kit according to the manufacturer's instructions.

### Immunochemical Methods

#### Elisa

Screening of antisera spent medium and ascites for the presence of antibodies against *Cronobacter *was performed by an indirect non-competitive ELISA. Flat-bottom 96 well plates were coated with 0.1 ml of (10^8 ^heat-killed cells ml^-1^) of whole cell antigen diluted in 0.05 M carbonate buffer (pH 9.6) overnight at 4°C. Alkaline phosphatase-conjugate goat anti-mouse immunoglobulin and p-nitrophenyl phosphate were used as secondary antibodies and substrate, respectively.

#### Additive index elisa

Additive index ELISA was performed on paired MAbs as described by Friguet et al., [29]. An additive index for each pair of MAbs was calculated according to the formula {[2*A*_1+2_/(*A*_1 _+ *A*_2_)] - 1} × 100, where *A*_1_, *A*_2_, and *A*_1+2 _are absorbance values with antibody 1 alone, antibody 2 alone, and the two antibodies together, respectively.

#### Gel electrophoresis

Profiles of *Cronobacter *OMPs were examined using SDS-PAGE following the method described by Laemmli [30]. The runs were performed in 4% stacking and 12.5% separating gels. Equal concentrations of *Cronobacter *OMPs (20 μg well^-1^) were mixed with sample buffer at a ratio of 1:5, boiled for 5 min and loaded (approx. 20 μl/lane). Gels were either stained with 1% (w/v) Coommassie Brilliant Blue G-250 or used for immunoblotting. Likewise, LPS preparations from *Cronobacter *were examined using Deoxycholate-polyacrylamide gel electrophoresis (DOC-PAGE) following the method described by Reuhs et al., [31]. Briefly, the runs were performed using 4% (v/v) stacking and 12.5% (v/v) separating gels. Equal concentrations of *Cronobacter *LPS (5 μg well^-1^) were mixed with sample buffer [2 ml of 22.7% (w/v) Tris-base solution; 1 ml of 50% (v/v) glycerol; 1 ml of 1% (w/v) bromophenol blue and 6 ml distilled water] at a ratio of 1:5. The gels were pre-run in DOC-electrophoresis buffer (Tris-base, 4.5 g; glycine, 21.7 g; 2.5 g sodium deoxycholate, pH adjusted to 8.3 and volume adjusted to 1 liter) for 10 min at 80 volts before loading the samples. Samples were run in the same buffer at 80 and 120 volts for the stacking and separating gels, respectively. Upon completion, gels were either stained using the PageSilver™ silver staining kit (Fermentas) or were used for immunoblotting. Band sizes for both gels and blots were estimated using the BioRad Quantity One 1-D Analysis Software.

#### Immunoblotting

Immediately after completing the electrophoresis run, OMPs and LPS were transferred to nitrocellulose (NC) membranes according to Harlow and Lane [28] with some modifications. Gels and NC membranes were soaked in Tris-glycine transfer buffer (10% [v/v] methanol, 24 mM Tris base, 194 mM glycine) for 15 min. Separated OMPs and LPSs were transferred onto NC using a mini-transblot cell (Bio-Rad). The membranes were blocked with 3% (w/v) BSA in Tris Buffered Saline (TBS) containing Tween 20 (0.05% v/v). NC membranes were then incubated with affinity purified MAbs (2 μg ml^-1^) diluted in 0.15 M TBS buffer containing 1% (w/v) BSA with gentle shaking for 1 h. Membranes were then developed with goat anti-mouse-HRP in 0.15 M TBS buffer containing 1% (w/v) BSA and a diaminobenzidine (DAB) substrate solution. Color development was stopped by rinsing the membranes with distilled water.

#### Protein sequencing and identification

Extracted OMPs were separated on SDS-PAGE gels and probed with anti-OMP monoclonal antibodies. Immunoblot-positive bands were cut with sterile sharp scalpel and immersed in 1% acetic acid solution. Protein sequencing was performed using the MALDI-TOF technology at the Proteomics and Mass Spectrometry Facility at Purdue University (West Lafayette, Indiana, USA).

#### Dot blot assay

Dot blotting was performed as described by Jaradat and Zawistowski [23]. One microliter of heat-killed *Cronobacter *whole-cell suspension (10^8 ^cells ml^-1^) was spotted on the NC membranes, allowed to air dry for 30 min and incubated in 5% (w/v) NaOH or in 38% (v/v) HCl for 10 s or left untreated. Immunoblotting was performed as described above.

#### Immunoelectron microscopy

Immunolabeling was performed essentially as described by Jaradat and Zawistowski [23] with modifications. Briefly, 5 μl of bacterial suspension in distilled water (5 × 10^8 ^CFU ml^-1^) were placed on formvar-coated copper grids. After air-drying for 2 h at room temperature, the grids were blocked with PBS containing 3% (w/v) BSA for 30 min at 37°C. To expose antigens on bacteria, grids were incubated with 0.1 M NaOH or 0.1 M HCl for 2 h, washed with water and incubated with purified MAb solution at 37°C. Grids were then incubated with colloidal gold (18 nm)-conjugate anti-mouse IgG diluted at 1:50 in dilution buffer (0.02 M Tris, 150 mM NaCl, 0.1% [w/v] BSA, 0.005% [v/v] Tween 20, 0.4% [w/v] gelatin [pH 9]) for 20 h at room temperature. Grids were washed 6 times with water and viewed with a Zeiss Transmission Electron Microscope at various magnifications.

#### Animal use

Animals used for immunization and production of monoclonal antibodies were cared for according to the Animal Care and Use Committee (ACUC), Jordan University of Science and Technology.

## Results

**Figure 1:**
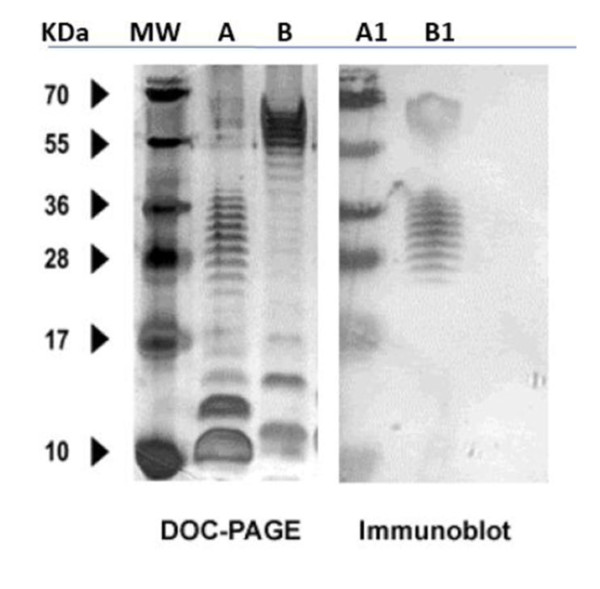
**DOC-PAGE (left panel) and immunoblotting (right panel) for LPS extracted from *C. muytjensii *ATCC 51329 (lanes A and A1) and *E. coli *(lanes B and B1)**. Blots were probed with mouse antisera collected after immunization with LPS preparation from *C. muytjensii *ATCC 51329.

Two approaches were attempted to produce monoclonal antibodies specific to *Cronobacter *spp.: one group of mice was immunized with heat-killed *C. muytjensii *ATCC 51329, while the other group was immunized with LPS purified from *C. muytjensii *ATCC 51329. Repeated immunization with the LPS produced a good antibody response as judged from both ELISA and immunoblotting results using antisera from LPS-immunized mice which revealed the characteristic ladder pattern of LPS (Figure 1). However, none of the two immunization protocols resulted in a stable hybridoma producing anti-LPS antibodies. Nevertheless, mice immunized with heat-killed cells responded well yielding a high titer after 5 injections. Consequently, mice from this group were sacrificed and two fusions were performed yielding over 500 hybridomas of which approximately 180 clones were positive upon initial screening and were cloned 3 times by limiting dilution [32-34]. Of these, only 5 stable hybridomas secreted antibodies against *Cronobacter *spp. Four of the hybridomas were of IgG type (A1, B5, 2C2 and C5), while the last hybridoma (A4) was of the IgM class. The avidity of the MAbs to their epitopes was determined by ELISA. The titration curve for all protein G-column purified MAbs, except for A4, revealed that MAb-2C2 had the highest avidity followed by C5, B5 and A1 having the lowest. MAb A4, an IgM, was not tested as it was not purified by Protein G column affinity chromatography.

### Specificity of the monoclonal antibodies

The specificity of the MAbs was determined by non-competitive ELISA with various heat- killed bacteria belonging to *Cronobacter *and non-*Cronobacter *spp. In general, all MAbs reacted with both *Cronobacter *and non-*Cronobacter *spp. with higher titers generally obtained for *Cronobacter *spp. (Titer of 3200 *Cronobacter *versus 400 for some non-*Cronobacter*). Nevertheless, some non-*Cronobacter *spp. also gave titers comparable to those obtained for *Cronobacter *(Titer 3200). The binding affinities varied among the four MAbs with MAbs 2C2 and C5 gave titers of 3200 against almost all the heat-killed *Cronobacter *strains tested, whereas MAbs A1 and B5 had titers ranging between 800 to more than 6400.

In addition to ELISA, the antigenic specificity of all purified MAbs was tested against OMPs extracted from 12 *Cronobacter *and 6 non -*Cronobacter *strains by SDS-PAGE followed by immunoblotting.

**Figure 2:**
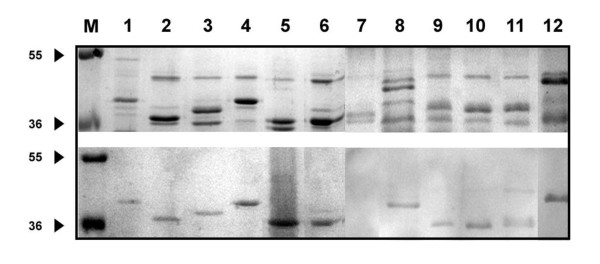
**SDS-PAGE (Upper) and Immunoblot (Lower) of OMPs extracted from different *Cronobacter *strains probed with MAb 2C2**. M: Molecular weight marker; 1: *C. muytjensii *ATCC 51329; 2: C4; 3: C6; 4: C13; 5: Jor93; 6: Jor170; 7: Jor44; 8: Jor112; 9: Jor146_A_; 10: Jor146_B_; 11: Jor149; 12: Jor160_A_.

**Figure 3:**
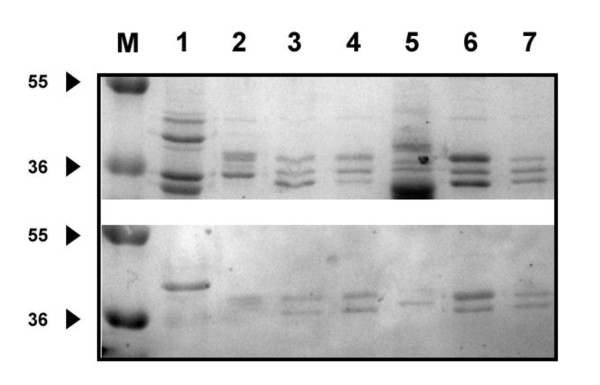
**SDS-PAGE (Upper) and Immunoblot (Lower) of OMPs extracted from different Non-*Cronobacter *strains probed with MAb 2C2**. M: Molecular weight marker; 1: *C. muytjensii *ATCC 51329; 2: *Citrobacter freundii*; 3: *E. coli*; 4: *Pseudomonas aeruginosa*; 5: *Listeria ivanovii*; 6: *Salmonella*; 7: *Shigella sonnii*

**Figure 4:**
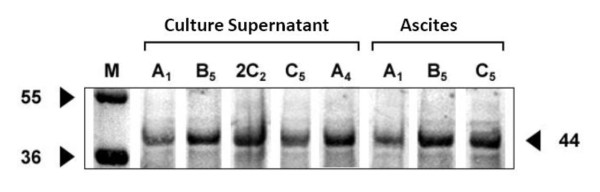
**Immunoblot for SDS-PAGE gel of OMP extracted from *C. muytjensii *ATCC 51329 and probed with all MAbs**. M: Molecular weight marker

SDS-PAGE profiles of both *Cronobacter *and non-*Cronobacter *revealed the presence of several proteins with molecular weights ranging from 12 to 100 kDa (data not shown) with the majority of OMPs profiles contained 3 to 5 major proteins having molecular weights between 34 and 55 kDa. Upon immunoprobing, all MAbs produced similar reaction profile of proteins with molecular weights between 36 to 49 kDa with only one major band for all *Cronobacter *OMP profiles except isolate Jor44 which did not exhibit any protein (Figure 2, lane 7, *C. sakazakii*, lower panel). In general, the 36 kDa protein was the most common among all *Cronobacter *OMP profiles. It was detected by immnunoblotting in 5 out of the 12 tested *Cronobacter *strains (Figure 2, lower panel; lanes, 5, 6, 9, 10, 11; *C. sakazakii, C. turicensis, C. sakazakii, C. sakazakii, C. muytjensii*, respectively), and a 42 kDa protein was detected in two *C. sakazakii *isolates (Figure 2, lower panel; lanes, 1 and 12), while a 41 kDa protein was detected in two isolates (*C. sakazakii*, and *C. muytjensii*, lower lanes 4 and 8 respectively). In addition, proteins of 37 and 39 kDa were detected each in one *C. sakazakii *isolate (Figure 2, lower panel; lanes 2 and 3, respectively). OMPs from a number of non-*Cronobacter *species were also tested for reactivity against the purified MAbs by immunoblotting. Unlike the single band pattern observed in *Cronobacter *OMP immunoblot profiles, the non-*Cronobacter *immunoblot profiles exhibited a double-band pattern corresponding to MW of 38 and 41 kDa (Figure 3 lower panel; lanes 3, 4, 6 and 7 representing strains; *E. coli, P. aeroginusa, Salmonella*, and *S. sonnii *respectively). In addition, *C. freundii *(Figure 3 lower panel; lane 2) exhibited only one protein corresponding to 40 kDa, while the only Gram-positive control strain (*L. ivanovii*, lane 5) exhibited the same two bands with a higher MW (39 and 42 kDa) than those which appeared in samples from the Gram-negative isolates. In order to determine which surface antigen the MAbs were bound to, OMPs and LPS extracted from *Cronobacter muytjensii *ATCC 51329 (strain used for immunization) were analyzed by SDS-PAGE, DOC-PAGE and immunoblotting. All MAbs (A1, B5, 2C2, C5 and A4) displayed strong and specific reaction against a 44 kDa OMP (Figure 4). Although these MAbs were produced from two different fusion experiments, they all reacted strongly and specifically against the same 44 kDa OMP. Furthermore, to compare the epitope specificity of all MAbs, additive index ELISA was conducted for each pair of the MAbs. All scores obtained were very low and fall in the range of 0 to 10 indicating that all MAbs were raised against the same epitope within the 44 kDa OMP.

### Protein identification by MALDI-TOF peptide sequencing

Representatives of the immunoblot-positive proteins were subjected to peptide sequencing and identification using MALDI-TOF Mass spectrometer. Table 2 shows the identified proteins by MALDI-TOF. The 44 kDa protein that was recognized by all the monoclonal antibodies in *C. sakazakii *appeared to be a novel protein that did not match with any identified protein thus was termed a hypothetical protein.

**Table 2:** Protein bands identified by MALDI-TOF mass spectrometer

Band	Strain	Predicted MW (kDa)	Protein annotation (NCBI database)	**Accession No**.	No. of peptides identified by MS/MS
1	160_A_(*C. sakazakii*)	42	Flagellar hook protein FlgE [*Shigella sonnei *Ss046]	gi|74311638	1
2	*Escherichia coli*	35	Outer membrane protein (porin) [*Escherichia coli *B171]	gi|75211632	5
3	*Escherichia coli*	38	Outer membrane protein A [*Escherichia coli *536]	gi|110641146	7
4	*Salmonella *CIP	35	Outer membrane protein (porin) nmpc precursor [*Escherichia coli *CFT073]	gi|26247429	6
5	*Salmonella *CIP	38	Outer membrane protein A [*Escherichia coli *536]	gi|110641146	8
6	C13(*C. sakazakii*)	42	P COG3203: Outer membrane protein (porin)[*Escherichia coli *101-1]	gi|83587007	1
7	112 (*C. muytjensii*)	40	Outer membrane protein F [*Escherichia coli *SMS-3-5]	gi|170682361	1
8	146_A _(*C. sakazakii*)	35	Hypothetical protein ESA_02413 [*Enterobacter sakazakii *ATCC BAA-894]	gi|156934579	8
9	*C. muytjensii *ATCC 51329	44	Hypothetical protein ESA_03699 [*Enterobacter sakazakii *ATCC BAA-894]	gi|156935823	3

In addition, the 35 kDa protein identified in the *Cronobacter *isolate 146_A _also appeared to be a novel protein termed a hypothetical protein that did not match with any known protein sequence deposited in the protein sequence bank (Table 2). Two *Cronobacter *isolates (160_A _and C13) exhibited a 42 kDa protein with identity as a flagellar hook protein FlgE and an outer membrane porin protein in the two isolates respectively. Further, a 40 kDa protein was recognized in *Cronobacter *isolate 112, and appeared to be an outer membrane protein F which is similar to an outer membrane protein F in *E. coli*. Both *E. coli *and *Salmonella *contained another similar protein with a MW of 38 kDa and was identified as an outer membrane protein A. In addition, both exhibited a 35 kDa porin protein yet appeared to be somewhat different.

### Effect of different treatments of antigens on MAbs binding affinity

**Figure 5:**
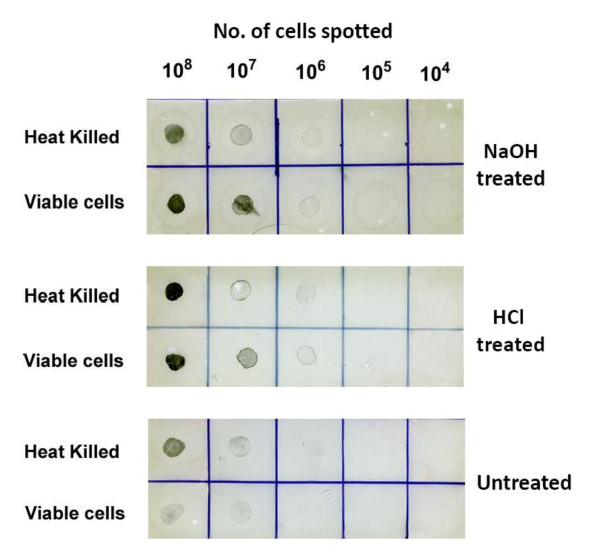
**Dot blot assay of whole cells of *C. muytjensii *ATCC 51329 at different concentrations of live or heat-killed**. Upper panel, cells treated with 5% NaOH for 10 s, middle panel cells were treated with 38% HCl for 10 s and lower panel, cells were left untreated. All blots were probed with MAb 2C2.

**Figure 6:**
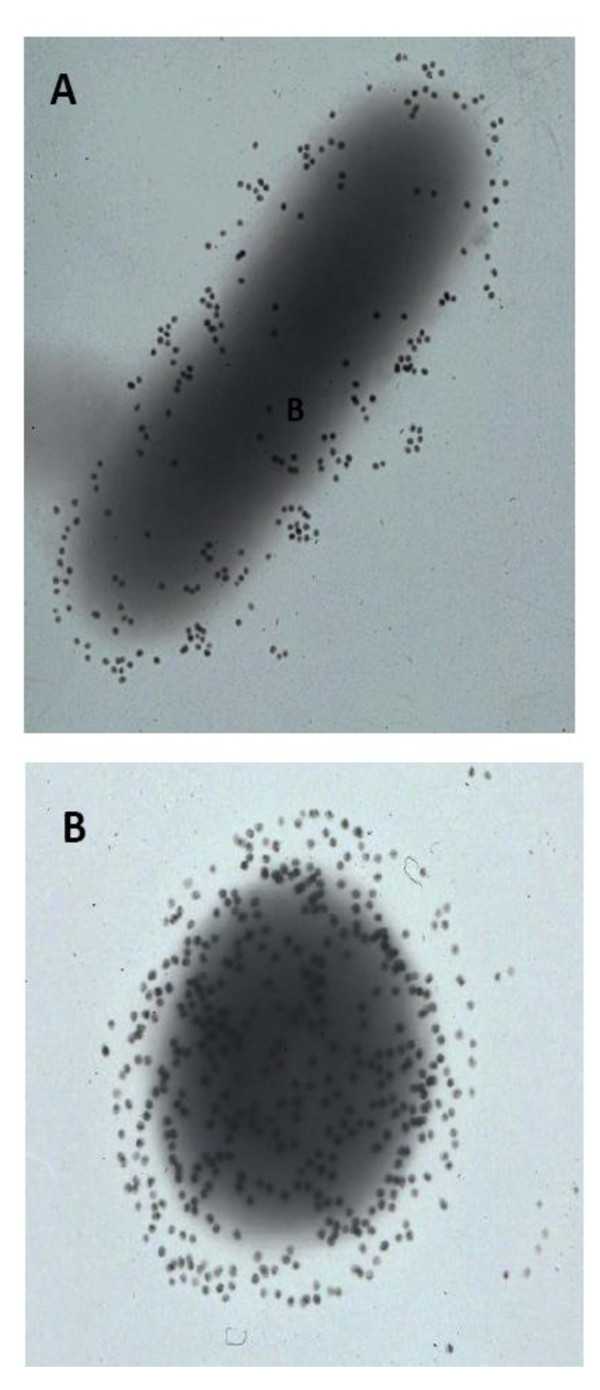
**Transmission electron micrographs of *C. muytjensii *ATCC 51329 treated with 0.1 N NaOH A, or 0.1 N HCl B and probed with MAb 2C2 followed by goat anti-mouse Ig conjugated to 18 nm gold spheres**. Magnification × 50,000.

To gain insights about the nature of the binding between the MAbs and their target epitopes, ELISA and Dot-blot were carried out using different antigens (OMPs, heat killed bacterial cells, LPS) which were subjected to different treatments (acid, alkaline, denaturing agents and heat) (Figure 5). Acid and base-treatments of whole cell antigens resulted in an increase in the binding affinity between the MAbs and those antigens. These results were confirmed by immunoelectron microscopy. *Cronobacter muytjensii *ATCC 51329 cells displayed intense colloidal gold labeling after reaction with MAb 2C2 (similar results were obtained with the other MAbs) (Figure 6).

Finally, to determine whether the MAbs recognized sequential (Linear) or conformational epitopes, OMPs were either left intact or denatured by 1% (w/v) SDS and boiled for 5 min and then used as antigens for ELISA. The magnitude of binding of MAbs to antigens was higher for untreated OMPs than the denatured proteins (Table 3). This indicates that, the epitope is conformational and loses its recognition sites once denatured.

**Table 3:** Reactivity of MAbs with different types of treated and untreated antigens as assessed by ELISA.

Type of antigen **	Treatment	Absorbance (405 nm) ± SD *			
		
		A1	B5	2C2	C5
OMP	None	1.375 ± 0.20	0.720 ± 0.15	1.234 ± 0.58	1.481 ± 0.12
OMP	1% SDS + Boiling for 5 min	0.958 ± 0.07	0.492 ± 0.04	0.562 ± 0.08	0.901 ± 0.08
WC	None	1.365 ± 0.08	0.565 ± 0.07	0.725 ± 0.08	0.835 ± 0.03
WC	Heat	1.156 ± 0.16	0.423 ± 0.08	0.782 ± 0.03	1.026 ± 0.19
LPS	None	0.553 ± 0.08	0.454 ± 0.04	0.425 ± 0.09	0.531 ± 0.04
None	None	0.477 ± 0.05	0.469 ± 0.24	0.520 ± 0.07	0.412 ± 0.17

OMP: outer membrane protein; WC: whole cell; LPS: Lipopolysaccharides, SD: Standard deviation.

* Absorbance represents the average of two readings

** All antigens were prepared from *C. muytjensii *ATCC 51329

## Discussion

Antibodies against surface antigens of pathogens aid not only in characterization but also in their classification [35]. In this study monoclonal antibodies were produced against outer membrane proteins of *Cronobacter muytjensii*. However, we were unable to produce antibodies against LPS. Inability to produce stable hybridomas against LPS could be attributed to the simplicity of the LPS structure which is a linear unbranched chain of repeating polysaccharide units as reported by MacLean et al., [7]. The linearity of the structure was probably responsible for the inability to elicit a significant immune response which was reflected on the inability to produce monoclonal antibodies against LPS of this strain. Luk and Lindberg [36] initially failed to produce stable antibody-producing hybridomas against LPS of *Salmonella*. Later, they succeeded when they used whole bacterial cells coated with LPS as immunogen. Similarly, Jongh-Leuvenink et al., [37] and Jaradat and Zawistowski [23] were able to produce monoclonal antibodies against LPS of *Salmonella*. This could be due to differences in the nature of the structure and composition of LPS between *Salmonella *and *Cronobacter *spp. and even among different *Salmonella *serovars.

In this study, the anti-OMP antibodies were characterized for specificity and all 5 monoclonal antibodies not only reacted with *Cronobacter *species, but also recognized other *Enterobacteriaceae*. The low specificity indicates that the major outer membrane proteins in the family *Enterobacteriaceae *are perhaps well conserved as indicated by their antigenic cross-reactivity. The specificity of the monoclonal antibodies was further tested using SDS-PAGE and immunoblotting. The SDS-PAGE protein profiles for the OMPs observed in this study were similar to those of OMPs described by other researchers for other members of the *Enterobacteriaceae *[38,39]. Overall, most of the isolates contained OMP proteins with MW ranged from 34-55 kDa (Figure 2 upper panel) with majority of the isolates exhibiting proteins in the range of 36-49 kDa with the 49 kDa protein appeared in all *Cronobacter *species (Figure 2 upper panel). In contrast, the non-*Cronobacter *isolates (Figure 3) showed slightly different protein profiles among the *Enterobacteriaceae *members and even a slight shift in the tested Gram-positive strain, *L. ivanovii*. The cross-reactivity observed among all *Cronobacter *strains used in this study indicated that some of these OMPs share common and highly antigenic epitopes. These patterns of cross-reactivity of MAbs with OMPs from bacterial strains within the same species are commonly reported especially for members of the *Enterobacteriaceae *[38-42]. On the other hand, fewer studies have reported the production of anti-OMP MAbs within species that were non-cross reacting and exhibiting a high degree of specificity [43,44]. The reactivity of MAbs to OMP and the lack of any reactivity against LPS indicated that *Cronobacter *OMPs appeared to be more antigenic than their LPS. This observation coincides with several other reports in which it was demonstrated that OMPS were stronger immunogenes than LPS, and were responsible for producing antibodies with higher affinities [45,46].

All MAbs tested by immunoblotting against OMPs extracted from *C. muytjensii *ATTC 51329 were able to recognize a 44 kDa protein. This protein appears to contain a highly antigenic epitope capable of eliciting strong immune response in mice against the *Cronobacter *strain used in the immunization procedure. The identity of this protein was determined by MALDI-TOF MS to be a hypothetical outer membrane protein ESA_03699 [*Enterobacter sakazakii *ATCC BAA-894]. This protein appeared to be dominant in this particular strain and protruding to the surface making it highly accessible to the host immune system. The specific function of this protein is unknown but it would be of significant interest in future studies since it was not detected in other strains. Other proteins from *Cronobacter *and non-*Cronobacter (E. coli *and *Salmonella*) recognized by the MAbs were also sequenced and aligned against known protein sequences deposited in protein sequence banks. It appeared that most of these proteins are related in terms of their structure and probably function as most of them were outer membrane proteins. However, a 42 kDa protein that was identified in two different *Cronobacter *spp. appeared to be different both in structure and function as one appeared to be a flagellar protein (*Cronobacter *160_A_), while the second was identified as an outer membrane protein (*Cronobacter *C13). Further, as shown in Table 2 some of the proteins with the same MW (e.g 35 kDa) were identified in three different bacteria and each appeared to have a different peptide sequence and consequently different function yet share epitope similarity as they were all recognized by the same MAb indicating a similar function too. Interestingly, similar to the 44 kDa protein, the 35 kDa protein identified in *Cronobacter *isolate number 146_A _appeared as novel protein and was termed as a hypothetical protein ESA_02413 with unknown function. Further, a protein of 40 kDa MW was identified in *Cronobacter *isolate number 112 as an outer membrane protein F which is similar to a protein in other *E. coli *as revealed from the protein bank sequence (Table 2).

The findings in the current study provide an evidence of great similarity among *Cronobacter *spp. and the other members of *Enterobacteriaceae*. Such findings were comparable to several previous studies which reported similar cross reactivity among major OMPs in Gram negative bacteria and among members of the *Enterobacteriaceae *[38-42]. For example, monoclonal antibodies that recognized buried epitopes of the ompC from *Salmonella typhi *were shown to cross react with porins extracted from 13 species of *Enterobacteriaceae *[41]. In addition, it appeared that OMPs extracted from *Cronobacter *and non-*Cronobacter *spp. in this study shared similar epitopes. This was evident in the multiple proteins which were recognized by the same MAbs that appeared to be specific toward the 44 kDa OMP extracted from the *Cronobacter *strain used for immunization. Indeed, these results highlighted the heterogeneity of the OMPs in the *Cronobacter *isolates.

The effect of acid or base treatment on the reactivity of monoclonal antibodies to their antigens was investigated. Acid or base treatment increased binding affinity of the antibodies to *Cronobacter *cells. This might be due to an increase in the accessibility of MAbs to the surface protein antigens due to removal of some extracellular molecules and/or LPS that might have hindered the binding of MAbs to their target proteins in the case of whole bacterial cells. For example, LPS accounts for up to 70% of the outer monolayer [47]. Indeed, the masking effect of LPS against binding of antibodies to antigens has been reported and therefore it can not be under estimated [48]. These observations were further confirmed by immunoelectron transmission microscopy (Figure 6). When live untreated *Cronobacter *cells were probed with MAb 2C2, there was no binding to the primary antibodies and hence no gold particle labeling. However, when *Cronobacter *cells were treated with NaOH (Figure 6A) or HCl (Figure 6b), the antibodies appeared to have gained access to their target represented by increased labeling. In addition, the MAbs were shown to be bound more strongly to conformational rather than sequential (linear) epitopes highlighting the specificity of the MAbs to their epitopes as appeared in Table 3[41].

## Conclusions

To our knowledge, this is the first study that describes the production of monoclonal antibodies against whole cells of *C. muytjensii *with concomitant identification of the recognized proteins by MALDI-TOF spectrometry. All MAbs produced in this study were reactive against the whole cell antigen and *Cronobacter *OMPs. MAbs reacted with OMPs of molecular weight ranging between 36 and 49 kDa. However, none of the MAbs showed any reaction with LPS extracted from *Cronobacter*. All MAbs recognized conformational epitopes rather than sequential as it is evident from the decrease in their binding affinity to fully denatured OMP antigens. Moreover, all MAbs exhibited a high cross-reactivity against the whole cell antigen and OMPs from non-*Cronobacter*. As apparent from the MALDI-TOF protein identification, the overall results indicated that, the major OMPs found in the *Enterobacteriaceae *are sufficiently conserved thereby, promoting antigenic cross-reactivity between genera. Furthermore, the single-banding pattern and the high titers obtained in immunoblotting and ELISA for the *Cronobacter *strains indicated that the OMPs of closely related strains are more conserved compared with other genera evaluated. The results from this study can be of great help for possible vaccine production against this pathogen in infants and young children.

## Competing interests

The authors declare that they have no competing interests.

## Authors' contributions

ZJ secured the funding for the project, analyzed data and wrote the final manuscript, AR and QA conducted the experimental work and participated in drafting the initial manuscript, SJ helped in the experimental work and AB edited the manuscript and participated in data analysis. All authors have read and approved the final manuscript.
